# Feasibility of implementing recommendations to improve neglected tropical diseases surveillance and response in Kenya: a modified Delphi study

**DOI:** 10.1186/s12913-021-07075-y

**Published:** 2021-10-01

**Authors:** Arthur K. S. Ng’etich, Kuku Voyi, Clifford M. Mutero

**Affiliations:** 1 School of Health Systems and Public Health (SHSPH), University of Pretoria, Pretoria, South Africa; 2 University of Pretoria Institute for Sustainable Malaria Control (UP ISMC), University of Pretoria, Pretoria, South Africa; 3 International Centre of Insect Physiology and Ecology, Nairobi, Kenya

**Keywords:** Delphi survey, surveillance systems, neglected tropical diseases, feasibility, core functions, support functions and surveillance attributes

## Abstract

**Background:**

Effective health information systems (HIS) are critical towards achieving timely response to preventive chemotherapy neglected tropical diseases (PC-NTDs) and their eventual elimination. Strengthened HIS enable prompt case detection and effective response to halt disease transmission and prevent probable outbreaks. This study aimed to assess the importance and feasibility of implementing recommendations for improving surveillance core functions, support functions and surveillance attributes concerning PC-NTDs in Kenya.

**Methods:**

A descriptive web-based Delphi process comprising of two survey rounds was used to achieve group consensus on the importance of recommended actions and feasibility of their implementation. In the first round, participants were enrolled to complete a five-point likert-type self-administered electronic questionnaire comprising of 60 statements across 12 sub-domains on the importance of recommendations. In the second round, participants reappraised their responses following completion of a questionnaire with 56 rephrased statements on feasibility of implementing the recommendations to improve PC-NTDs surveillance and response. Data from both rounds were analysed using descriptive statistics and thematic analysis performed for the open-ended responses.

**Results:**

Sixty-two key stakeholders actively involved in surveillance and response activities in seven PC-NTDs endemic counties in Kenya were invited to participate. Of these, 50/62 completed the first round (81 % response rate) and 45/50 completed the second round (90 % response rate). Consensus was achieved (defined as > 70 % agreement) on the importance (93 %) of recommendation statements and feasibility (82 %) of implementing the important recommendations. Stakeholders agreed on the importance and feasibility of specific recommendations across the 12 sub-domains: case detection and registration, reporting, data analysis, feedback, epidemic preparedness and response, supervision, training, resources, simplicity, acceptability, stability and flexibility. However, there was lack of consensus on the feasibility of conducting routine data analysis, increasing supervision of surveillance activities at lower levels and retaining trained surveillance staff across all levels.

**Conclusions:**

Consensus among health stakeholders on implementation of the important and feasible recommendations will inform relevant strategies for strengthening specific surveillance system functions in view of PC-NTDs in Kenya.

**Supplementary Information:**

The online version contains supplementary material available at 10.1186/s12913-021-07075-y.

## Background

Public health surveillance systems are a critical component of health information systems (HIS) and involve systematic collection, analysis and interpretation of health data resulting in timely information dissemination to prompt appropriate response action [1]. In 1998, the integrated disease surveillance and response (IDSR) strategy was adopted by member states of the African region [2, 3]. This strategy was intended to harness and integrate common surveillance functions and resources, which were previously utilised through a single or vertical surveillance approach [2, 3]. In Kenya, the IDSR strategy was first adopted in the year 2000 and later in 2012 the revitalised IDSR guidelines were adopted [4]. Evidently, surveillance system assessments previously conducted in Africa have mostly put focus on diseases considered of a notifiable nature [5–8]. Consequently, there is insufficient documentary evidence on disease-specific recommendations to improve surveillance and response systems concerning other diseases targeted for elimination or eradication such as the neglected tropical diseases (NTDs). Neglected tropical diseases are a diverse group of infections mainly prevalent in tropical and sub-tropical conditions [9]. The insidious diseases contribute immensely to the burden of health conditions including irreversible blindness, hepatosplenomegaly and lymphadenopathy. In addition, NTDs are associated with disability, disfigurement and stigmatization [9–11].

Strengthened surveillance systems will facilitate disease elimination programmes by improving existing HIS with regard to case detection and public health response activities targeting disease transmission foci and outbreaks prevention [12]. Therefore, there is dire need to establish and implement novel surveillance approaches and strategic responses to transition from disease control to elimination [13]. This is in line with the fourth strategic priority of the Second Kenya National Strategic Plan for NTDs (2016–2020), the fifth strategic objective of the Kenya National Breaking Transmission Strategy for preventive chemotherapy neglected tropical diseases (PC-NTDs) (2019–2023) and the Expanded Special Project for Elimination of NTDs (ESPEN) [14–16]. A key intervention strategy to achieving sustainable NTDs control is through preventive chemotherapy (PC). This chemoprophylaxis intervention involves administering a single drug regimen, either alone or in combination with other drugs, as a public health control measure for targeted NTDs [17, 18]. The population-based nature of PC interventions is considered a pragmatic approach to achieving NTDs control given its reliance on safe drugs, which can be administered on a large-scale and on regular intervals by non-medical personnel [18].

In the Kenyan context, majority of the NTDs are amenable to PC and other basic sanitary interventions [14]. Essentially, effective control and prevention of NTDs is dependent on early detection and prompt action aided by effective surveillance and response systems [12, 13]. In the absence of reliable surveillance and response systems, targeted disease control is impeded since interventions may be implemented in areas where NTDs are uncommon or completely absent, which may result in unnecessary use of the limited resources [19]. The current study approach provides critical information to explore opportunities for strengthening surveillance activities for PC-NTDs and the existing information systems for evidence-based action. Effective implementation of recommendations is dependent on consensus among stakeholders on their acceptability and feasibility for implementation. The Delphi study approach has been used widely in various disciplines and is perceived effective for studies aiming to show agreement among a group of stakeholders. The process allows stakeholders to revise their opinions raised on an issue at a prior stage based on responses from their counterparts [20].

A shift in focus of NTD programmes from donor-funded agencies to local stakeholders to motivate country’s self-reliance to curb NTDs, underpins the importance of in-country perspectives and decision-making to achieve sustainable impact [21]. Sustainable NTDs control efforts remain elusive especially if little or no consideration is accorded to local stakeholders’ contributions to decision-making. NTDs policy agenda setting should prioritise engaging the relevant healthcare stakeholders in the affected regions [22]. In the current study, the term ‘stakeholders’ was used to describe individuals actively involved in making decisions regarding disease surveillance activities or members of the health management committees constituted at the sub national level. Therefore, the study aimed to assess feasibility for implementing recommendations to improve PC-NTDs surveillance and response activities based on health stakeholders’ perspectives.

## Methods

### Survey design

The Delphi technique is a scientific approach involving sequential surveys where a series of rounds are used to obtain individual expert opinions to establish consensus among participants [23, 24]. The structured process utilises sets of questionnaires or iterative rounds to collect information in order to reach group consensus [25]. Prior to subsequent rounds, participants are provided with analysed outcome of each initial around to assess the level of consensus [24]. The Delphi methodology provided an opportunity to gain in depth perspectives from a diverse group of key stakeholders – selected from multiple counties in Kenya endemic of PC-NTDs – to reach consensus on what they would consider feasible for implementation [24].

A two-round descriptive web-based Delphi survey was utilised in the current study. The factors assessed in the survey were retrieved from key findings of three previous studies [26–28]. The first study involved a systematic review of literature based on surveillance assessment studies, which identified priority recommendations to improve surveillance core functions, support functions and surveillance attributes in the African region (PROSPERO Registration number CRD42019124108). The reviewed studies assessed surveillance and response systems in African countries post-adoption of the revised IDSR guidelines from 2010 onwards [26]. The second and third studies involved assessment of surveillance core functions, support functions and surveillance system attributes to identify disease-specific recommendations to improve PC-NTDs surveillance and response based on health workers’ perspectives in Kenya [27, 28]. Critical recommendations derived from the systematic literature review and primary data studies were based on healthcare personnel perceptions in view of their involvement in disease surveillance and response activities. Consequently, assessing the importance and feasibility for implementing recommendations provided in the previous studies formed the basis for the current study.

**Fig. 1:**
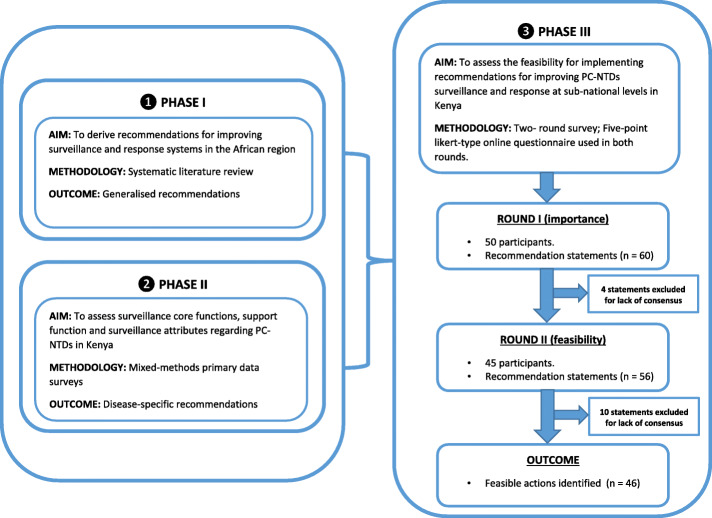
Schematic illustration of the Delphi study

Initially, three main domains (core functions, support functions and surveillance attributes) were identified. The three main domains were pre-defined from information retrieved from the Centres for Disease Control and Prevention (CDC) updated guidelines for evaluating public health surveillance systems [29]. Core functions are based upon indicators that measure disease surveillance system processes and outputs while support functions guide and facilitate implementation of core functions. On the other hand, surveillance system attributes are unique characteristics enabling the system to meet its objectives [29]. Twelve sub-domains emerged from the three main domains; (i) case detection, registration and confirmation, (ii) reporting, (iii) data analysis, (iv) feedback, (v) epidemic preparedness and response; (vi) supervision, (vii) training, (viii) resources, (ix) simplicity, (x) acceptability, (xi) stability, and (xii) flexibility. Recommendation statements were informed by findings from the three previous studies, which identified generalised recommendations to improve surveillance and response systems and disease-specific recommendations to improve surveillance core functions, support functions and surveillance attributes regarding PC-NTDs (Fig. 1) [26–28]. Similar recommendation actions derived from the studies were consolidated. This resulted to 60 recommendation statements, which were assessed using the Delphi survey. The 60 statements were grouped into the twelve sub-domains. In the first round, statements were phrased to elicit participants’ responses on the importance of recommendations while in the second phase the statements were re-phrased to prompt responses on feasibility for implementing the important recommended actions.

### Study setting and participant selection

The survey involved stakeholders selected from NTDs endemic regions in Kenya. Kenya comprises of 47 counties that are endemic of at least one or more NTDs. However, stakeholders were enrolled from seven specific regions including Baringo, West Pokot, Narok, Kilifi, Kwale, Embu and Kitui counties that are co-endemic of at least three or more PC-NTDs. Selection of regions co-endemic of three or more PC-NTDs was aimed at obtaining stakeholders’ perspectives considering the integrated disease surveillance and response approach [4]. Furthermore, the seven counties were purposively selected to provide an adequate geographic representation of three NTD endemic regions in Kenya; Rift Valley (Baringo, West Pokot and Narok counties), Coastal (Kilifi and Kwale counties), and Eastern (Embu and Kitui counties) regions. These counties also represent regions with the highest endemicity of PC-NTDs according to data retrieved from the Second National Strategic Plan for NTDs in Kenya [14]. The PC-NTDs of focus in the current study included Lymphatic Filariasis, Schistosomiasis, Trachoma and Soil Transmitted Helminths.

The survey involved 50 health personnel responsible for overseeing surveillance and response activities at the sub-national level. The sub-national health system constitutes the county and sub-county levels with peripheral levels comprising of lower level facilities (dispensaries) and community health units [30]. Study participants were considered key implementers of disease surveillance-related activities and were purposely selected from the seven PC-NTDs endemic counties in Kenya. The participants included directors of health, NTDs coordinators, disease surveillance coordinators, health records information personnel and public health personnel both at the county and sub-county levels.

### Implementation of the survey

Due to the Coronavirus disease (COVID-19) pandemic situation and considering precautionary measures recommended by the Ministry of Health (MOH) in Kenya, an electronic web-based self-administered questionnaires was designed using Google forms and data was securely transferred from the forms to Google spreadsheets. The five-point likert scale online questionnaires were used to obtain data from the participants provided as supplementary files (Supplementary file 1 & 2). Response to the statements comprised of *strongly agree, agree, neither agree nor disagree, disagree* and *strongly disagree* with provision of a *do not know* option. Consensus was defined as > 70 % of participants either “agreeing” or “strongly agreeing” with the recommendation statements in each of the two rounds. The questionnaires had an additional comment section to elicit open responses from participants having further information not captured in the likert-scale statements. Questionnaire used in the first round consisted of 12 sub-categories with 60 statements. In the second round, statements considered important by participants were retained from the first round and rephrased. The questionnaire used in the first round comprised of statements phrased in a manner to prompt responses from participants regarding importance of the recommendation statements. In the second round, the statements were rephrased to elicit participants’ opinions on the feasibility for implementing important recommendations at the sub-national level.

An initial first round involved administering the online survey tool to selected stakeholders responsible for overseeing and making decisions on surveillance and response activities in NTD endemic regions in Kenya. Their responses on importance of recommendation statements were obtained, analysed and the statements rephrased to assess feasibility for implementing important recommendations. The final second round involved administering the revised web-based tool and providing participants with the outcome report of the previous first round. Priority was accorded to county health stakeholders’ perspectives since they are the key decision makers on health system functioning at the sub-national level, which is a devolved function overseen by county governments in Kenya [14]. The initial purposive sample of 62 participants were invited via email or WhatsApp to participate in the Delphi survey. In the first round, an internet link to the electronic form was distributed via the same channels for participants to complete the online questionnaire, following which quantitative analysis of the responses using frequencies and percentages in between the two rounds was undertaken to ascertain the degree of disagreement or agreement with the recommendation statements. A comment section was provided in the questionnaires to solicit open responses from the stakeholders on the statements in both rounds of the survey. The participants who completed the first round were further invited via email or WhatsApp to participate in the second round. Up to three weekly email or WhatsApp message reminders were sent to participants who failed to respond in the second round. Invitation to participate in the first round was sent on June 15, 2020 and the final round was conducted on July 6, 2020.

### Data analysis

Exported data from the Google spreadsheets were analysed using Stata Version 14.0 (College Station, 77,845 Texas USA). Descriptive statistics were used to summarise categorical variables and frequency distributions were described for participant responses measured on the five-point likert scale. Reliability and coherence between items in questionnaires used in both rounds were independently tested using Cronbach’s Alpha coefficient. The calculated coefficients ranged from 0.81 to 0.95 indicating sufficient reliability and inter-item correlation. The final questionnaires used in the first and second round comprised of recommendation statements with an average of five questions in each sub-category. All questionnaire items were retained and none was excluded following factor analysis, based on the principal component analysis, to ascertain the statements relevance as constructs in each of the sub-domains. A level of consensus of above 70 % is considered appropriate for Delphi studies [31, 32]. Two consensus scenarios were described, the first, which excluded the ‘neither agree nor disagree’ responses in the analysis. The second scenario involved conducting sensitivity analysis whereby responses of participants who indicated ‘neither agree nor disagree’ were combined with responses of those participants who were in agreement with the statements. This determined the overall consensus on importance and feasibility of implementing the recommendations. Open responses from participants were analysed thematically by deriving relevant sub-themes based on each of the pre-defined sub-domains.

### Ethical considerations

Approval for the study was obtained from the Faculty of Health Sciences Research Ethics Committee of the University of Pretoria in South Africa (Ethics Reference No: 27/2018) and the Institutional Research and Ethics Committee of Moi University/Moi Teaching and Referral Hospital in Kenya (Formal Approval No: IREC 2099). Additionally, the National Commission for Science, Technology and Innovation (NACOSTI) provided research authorisation to undertake the study in accordance with relevant guidelines and regulations (Reference No: NACOSTI/P/18/62,894/21,393). Permission was granted by the MOH, county health authorities and written informed consent was obtained from all study participants.

## Results

Sixty-two participants were recruited in the first round of the survey and a response rate of 81 % was obtained. The final sample size was 50 participants following exclusion of those who did not consent (n = 5) and those who were unavailable (n = 7) to participate in the study. The median number of years of work experience for participants in their current designation was 10 years (IQR: 8–12 years). Majority (64 %) of participants were members of the County Health Management Committee (CHMC) with 36 % being part of the Sub-County Health Management Committee (SCHMC). The second round of the Delphi survey involved 45 participants translating to a response rate of 90 % since five out of the 50 participants in the previous round were unavailable to participate in the second round. The stakeholders were grouped into various areas of work including health management (county directors of health); disease surveillance (county and sub-county disease surveillance and response coordinators and NTDs coordinators); public health (sub county public health personnel) and health information (county and sub-county health records and information personnel) (Table 1). The preponderance of participants reached agreement on the importance and feasibility of implementing recommendations to improve surveillance and response to PC-NTDs at the sub-national level. Out of the 60 statements in 12 sub-domains in the first round of the survey, stakeholders reached consensus on importance of 56 upon 60 (93 %) recommendation statements (Table 2). Furthermore, in the second round, participants reached consensus on the feasibility of implementing 46 upon 56 (82 %) important recommendation statements (Table 3).

**Table 1:** Socio-demographic characteristics of participants

Characteristic	n (%)
**Regions**	
Coast (Kwale and Kilifi)	20 (40 %)
Rift Valley (Baringo, West Pokot and Narok)	18 (36 %)
Eastern (Kitui and Embu)	12 (24 %)
**Age of participants**	
31–40 years	20 (40 %)
41–50 years	21 (42 %)
> 50 years	9 (18 %)
**Gender of participants**	
Female	14 (28 %)
Male	36 (72 %)
**Areas of work responsibility**	
Health management	3 (6 %)
Disease surveillance	19 (38 %)
Public health	14 (28 %)
Health information	14 (28 %)
**Health management committee level**	
Sub county	18 (36 %)
County	32 (64 %)

**Table 2:** Participants’ consensus status on the importance of recommendation statements (N = 50)

Domains	Sub-domains	Statements	Consensus	Non-consensus	Neutral
			**n (%)**	**n (%)**	**n (%)**
**Core Functions**	Case detection, registration and confirmation	Need to update surveillance guidelines currently in use at the sub-national level	49 (98)	-	1 (2)
		Need to update the available PC-NTDs case definitions currently in use at the sub-national level	41 (82)	3 (6)	6 (12)
		Training is required on practical application of the available PC-NTDs case definitions	50 (100)	-	-
		Availing case registers specific for registering PC-NTD cases is necessary	35 (70)*	4 (8)	11 (22)
		All PC-NTDs need confirmation at the lower surveillance levels	33 (66)*	4 (8)	13 (26)
		Increased number of laboratories at lower surveillance levels is required to improve PC-NTDs case confirmation capacity	45 (90)	2 (4)	3 (6)
		Need to properly equip laboratories at the health facility level to improve PC-NTDs case confirmation	49 (98)	-	1 (2)
		Need to provide an adequate number of skilled laboratory health personnel for effective confirmation of PC-NTDs	50 (100)	-	-
	Reporting	Need to ensure reporting forms are always readily available in all surveillance levels	50 (100)	-	-
		Need to avail updated reporting guidelines at the sub-national level	49 (98)	-	1 (2)
		Need to list all PC-NTDs in the existing reporting forms to improve surveillance data capture	45 (90)	-	5 (10)
		Immediate reporting of PC-NTD cases is required to improve planned response actions	44 (88)	2 (4)	4 (8)
		Need to adopt electronic reporting tools to improve transmission of PC-NTDs surveillance data to the next level	50 (100)	-	-
		Frequent training on reporting PC-NTDs using existing reporting forms is required	50 (100)	-	-
		Need to allocate adequate time for surveillance reports preparation and submission to the next levels	49 (98)	-	1 (2)
	Data Analysis	Need to enhance PC-NTDs surveillance data analysis at the sub-national level	49 (98)	-	1 (2)
		Analysis of PC-NTDs surveillance data should be conducted on a routine-basis	42 (84)	4 (8)	4 (8)
		Trend analysis of PC-NTDs reported cases should be undertaken periodically	45 (90)	-	5 (10)
		Enhanced training on PC-NTDs surveillance data analysis is required	50 (100)	-	-
		Clearly formulated PC-NTDs action thresholds are required	50 (100)	-	-
		Need to provide adequate data analysis tools and equipment	50 (100)	-	-
	Feedback	Need to improve feedback on PC-NTDs surveillance data at the sub-national level	50 (100)	-	-
		Need for timely feedback on PC-NTDs surveillance data reported to the next level	50 (100)	-	-
		Regular feedback on reported PC-NTDs surveillance data is required	47 (94)	-	3 (6)
		Need to adapt improved electronic feedback mechanisms	49 (98)	-	1 (2)
		Increased feedback on PC-NTDs to lower surveillance levels is required	46 (92)	-	4 (8)
	Epidemic preparedness and response	Updated PC-NTDs outbreak preparedness and response protocols are required	50 (100)	-	-
		Need for outbreak response teams to be well-constituted to respond to probable PC-NTDs outbreaks	47 (94)	1 (2)	2 (4)
		Need for emergency supplies to adequately respond to probable PC-NTDs outbreaks	50 (100)	-	-
		Regular training on PC-NTDs outbreak preparedness and response is required	50 (100)	-	-
**Support Functions**	Supervision	Need to enhance supervision of PC-NTDs surveillance activities at the sub-national level	50 (100)	-	-
		Regular supervision of PC-NTDs surveillance activities undertaken at the lower levels is required	46 (92)	-	4 (8)
		Formulation of supervisory schedules for PC-NTDs surveillance activities is necessary	45 (90)	-	5 (10)
		Increased frequency of supervisory visits at the lower surveillance levels is required	42 (84)	1 (2)	7 (14)
		Training and sensitisation of all health workers regarding supervisory activities is required	46 (92)	2 (4)	2 (4)
		Need for properly constituted supervisory teams to adequately supervise PC-NTDs surveillance activities	50 (100)	-	-
		Need for adequate resource provision to support supervision of PC-NTDs surveillance activities	49 (98)	-	1 (2)
		Need for increased participation of the community levels to support supervision of PC-NTDs surveillance activities	49 (98)	-	1 (2)
	Training	Need for training on disease surveillance conducted at sub-national level to focus on PC-NTDs	49 (98)	-	1 (2)
		Regular training specifically on PC-NTDs surveillance activities is necessary	50 (100)	-	-
		All health workers need to be involved in training on PC-NTDs surveillance activities	35 (70)*	3 (6)	12 (24)
		Availing adequate training materials and equipment is necessary across all surveillance levels	50 (100)	-	-
		Need to retain trained surveillance staff across all surveillance levels	50 (100)	-	-
	Resources	Increased funding is required to support PC-NTDs surveillance activities	50 (100)	-	-
		Availing electronic communication equipment for transmission of PC-NTDs surveillance data is required	50 (100)	-	-
		Improved transport and logistical support is necessary to facilitate PC-NTDs surveillance activities	50 (100)	-	-
		Increasing the number of health workers involved in PC-NTDs surveillance activities is necessary	41 (82)	5 (10)	4 (8)
		Increasing the number of designated surveillance focal persons is required	33 (66)*	6 (12)	11 (22)
		Need for improved telecommunication channels to support transmission of surveillance data	49 (98)	1 (2)	-
		Need for improved means of transportation to facilitate surveillance activities	50 (100)	-	-
**Attribute Functions**	Simplicity	Simplification of existing guidelines for completing reporting forms is required	46 (92)	-	4 (8)
		Need to simplify available forms to ease reporting of PC-NTDs	36 (72)	-	14 (28)
		Need to simplify PC-NTDs case definitions to ease application	42 (84)	-	8 (16)
		Need to simplify methods for PC-NTDs surveillance data collection and analysis	44 (88)	-	6 (12)
	Acceptability	Need for the health managers to support PC-NTDs surveillance activities in the region	50 (100)	-	-
		PC-NTDs need to be considered of public health importance in the region	50 (100)	-	-
	Stability	Need for challenges facing PC-NTDs surveillance activities to be addressed with minimal delays	50 (100)	-	-
		Sufficient resources to support PC-NTDs surveillance activities are required	50 (100)	-	-
	Flexibility	Need for existing surveillance systems to be well adapted to reporting all PC-NTDs in the region	50 (100)	-	-
		Existing surveillance systems need to adapt easily to changes in PC-NTDs information needs	50 (100)	-	-

* - statements not achieving the > 70 % consensus threshold

**Table 3:** Participants’ consensus status on the feasibility of implementing recommendation statements (N = 45)

Domain	Sub-domains	Statements	Consensus	Non-consensus	Neutral
			**n (%)**	**n (%)**	**n (%)**
**Core Functions**	Case detection, registration and confirmation	It is feasible to update surveillance guidelines currently in use at the sub-national level	50 (100)	-	-
		It is feasible to update the available PC-NTDs case definitions currently in use at the sub-national level	37 (82)	-	8 (18)
		It is feasible to train all health workers on the application of available PC-NTDs case definitions	31 (69)*	4 (9)	10 (22)
		It is feasible to increase the number of laboratories at lower surveillance levels to improve PC-NTDs case confirmation capacity	16 (35)*	3 (7)	26 (58)
		It is feasible to fully equip laboratories at the health facility level to improve PC-NTDs case confirmation	32 (71)	3 (7)	10 (22)
		It is feasible to provide an adequate number of skilled laboratory health personnel for effective confirmation of PC-NTDs	39 (87)	-	6 (13)
	Reporting	It is feasible to ensure reporting forms are always readily available in all surveillance levels	45 (100)	-	-
		It is feasible to avail updated reporting guidelines at the sub-national level	44 (98)	-	1 (2)
		It is feasible to list all PC-NTDs in the existing reporting forms to improve surveillance data capture	25 (55)*	3 (7)	17 (38)
		It is feasible to ensure immediate reporting of PC-NTD cases to improve planned response actions	30 (66)*	2 (4)	13 (29)
		It is feasible to adopt electronic reporting tools to improve transmission of PC-NTDs surveillance data to the next level	45 (100)	-	-
		It is feasible to offer frequent training on reporting PC-NTDs using existing reporting forms	44 (98)	1 (2)	-
		It is feasible to allocate adequate time for surveillance reports preparation and submission to the next levels	45 (100)	-	-
	Data Analysis	It is feasible to enhance PC-NTDs surveillance data analysis at the sub-national level	42 (93)	-	3 (7)
		It is feasible for analysis of PC-NTDs surveillance data to be conducted on a routine-basis	14 (31)*	22 (49)	9 (20)
		It is feasible to undertake trend analysis of PC-NTDs reported cases periodically	27 (60)*	3 (7)	15 (33)
		It feasible to enhance training on PC-NTDs surveillance data analysis	45 (100)	-	-
		It is feasible to have clearly formulated PC-NTDs action thresholds	43 (95)	-	2 (4)
		It is feasible to provide adequate data analysis tools and equipment to all surveillance levels	44 (98)	-	1 (2)
	Feedback	It is feasible to improve feedback on PC-NTDs surveillance data at the sub-national level	41 (91)	-	4 (9)
		It is feasible to provide timely feedback on PC-NTDs surveillance data reported to the next level	44 (97)	-	1 (2)
		It is feasible to provide regular feedback on reported PC-NTDs surveillance data	42 (93)	-	3 (7)
		It is feasible to adapt improved electronic feedback mechanisms	45 (100)	-	-
		It is feasible to increase feedback on PC-NTDs to lower surveillance levels	41 (91)	-	4 (9)
	Epidemic preparedness and response	It is feasible to provide all surveillance levels with updated PC-NTDs outbreak preparedness and response protocols	45 (100)	-	-
		It is feasible to have well constituted outbreak response teams to respond to probable PC-NTDs outbreaks	43 (95)	-	2 (4)
		It is feasible to provide adequate emergency supplies to respond to probable PC-NTDs outbreaks	41 (91)	-	4 (9)
		It is feasible to provide regular training on PC-NTDs outbreak preparedness and response	45 (100)	-	-
**Support Functions**	Supervision	It is feasible to enhance supervision of PC-NTDs surveillance activities at the sub-national level	41 (91)	-	4 (9)
		It is feasible to provide regular supervision of PC-NTDs surveillance activities undertaken at the lower levels	23 (51)*	2 (4)	20 (44)
		It is feasible to formulate supervisory schedules for PC-NTDs surveillance activities	45 (100)	-	-
		It is feasible to increase the frequency of supervisory visits to the lower surveillance levels	10 (22)*	20 (45)	15 (33)
		It is feasible to train and sensitise all health workers regarding supervisory activities	43 (95)	1 (2)	1 (2)
		It is feasible to properly constitute supervisory teams to adequately supervise PC-NTDs surveillance activities	45 (100)	-	-
		It is feasible to ensure there is adequate resource provision to support supervision of PC-NTDs surveillance activities	44 (98)	-	1 (2)
		It is feasible to increase community participation to support supervision of PC-NTDs surveillance activities	44 (98)	-	1 (2)
	Training	It is feasible to enhance PC-NTDs surveillance trainings conducted at the sub-national level	44 (97)	-	1 (2)
		It is feasible to provide regular training specifically on PC-NTDs surveillance activities	39 (87)	-	6 (13)
		It is feasible to avail adequate training materials and equipment across all surveillance levels	45 (100)	-	-
		It is feasible to retain trained surveillance staff across all surveillance levels	12 (27)*	24 (53)	9 (20)
	Resources	It is feasible to increase funding to support PC-NTDs surveillance activities	34 (75)	-	11 (24)
		It is feasible to provide electronic communication equipment for transmission of PC-NTDs surveillance data	45 (100)	-	-
		It is feasible to improve transport and logistical support to facilitate PC-NTDs surveillance activities	45 (100)	-	-
		It is feasible to increase the number of health workers involved in PC-NTDs surveillance activities	18 (40)*	2 (4)	25 (56)
		It is feasible to improve telecommunication channels to support transmission of surveillance data	45 (100)	-	-
		It is feasible to improve means of transportation to facilitate surveillance activities	45 (100)	-	-
**Attribute Functions**	Simplicity	It is feasible to simplify existing guidelines for completing reporting forms	45 (100)	-	-
		It is feasible to simplify available forms to ease reporting of PC-NTDs	45 (100)	-	-
		It is feasible to simplify PC-NTDs case definitions to ease application	37 (82)	-	8 (18)
		It is feasible to simplify methods for PC-NTDs surveillance data collection and analysis	45 (100)	-	-
	Acceptability	It is feasible for the health managers to support PC-NTDs surveillance activities in the region	45 (100)	-	-
		It is feasible to influence health workers’ perceptions on the public health importance of PC-NTDs in the region	43 (96)	-	2 (4)
	Stability	It is feasible to address challenges facing PC-NTDs surveillance activities with minimal delays	36 (80)	3 (7)	6 (13)
		It is feasible to avail sufficient resources to support PC-NTDs surveillance activities	44 (98)	1 (2)	-
	Flexibility	It is feasible for the existing surveillance systems to be well adapted to reporting all PC-NTDs in the region	32 (71)	2 (4)	11 (24)
		It is feasible for the existing surveillance systems to adapt easily to changes in PC-NTDs information needs	33 (73)	3 (7)	9 (20)

* - statements not achieving the > 70 % consensus threshold

### Surveillance core activities

Stakeholders agreed on the importance (98 %) and feasibility (100 %) of updating surveillance guidelines currently in use at the sub-national levels and were further in consensus (82 %) on the need to update the available standard case definitions for PC-NTDs. Further, participants agreed on the importance of training health workers on practical use of the standard case definitions. However, there was non-consensus (69 %) on the feasibility of training all health workers on use of PC-NTDs standard case definitions. There was further non-consensus on the importance (70 %) of providing case registers specific for registration of PC-NTDs cases.

*“Having in place case registers for registering PC-NTDs only may not be practical and cost effective since we have many other conditions to be registered and it would be easier to do this using one common register”* (Participant 5).

*“It is only feasible to issue facilities designated as treatment sites with the registers since they receive NTD patients more often compared to the other facilities*” (Participant 16).

*“Providing registers specific for NTDs to all levels might require additional resources which are currently unavailable”* (Participant 32).

Furthermore, participants disagreed (66 %) on the need to confirm all PC-NTDs cases at the lower surveillance levels. Nonetheless, participants reached consensus (90 %) on the importance of increasing the number laboratory facilities at the peripheral levels to confirm PC-NTDs, although there was non-consensus (35 %) on the feasibility of having additional laboratories at lower surveillance levels.

*“It will not be quite possible to always confirm all the NTDs due to low capacity of the lower levels to undertake case confirmation due to unavailability of laboratory facilities”* (Participant 4).

*“Confirmation of all PC-NTDs at the lower levels would require increased provision of laboratory facilities and equipment in the region to achieve this target”* (Participant 8).

*“Confirming all cases requires adequate supply of laboratory reagents and supplies that may not be the case for most of the lower levels”* (Participant 29).

There was agreement among stakeholders on the importance (98 %) and feasibility (71 %) of adequately equipping existing laboratories at the facility level and providing sufficient number of skilled laboratory personnel to enhance PC-NTDs case confirmation.

 Further findings showed agreement on the importance and feasibility of ensuring reporting forms are always readily available and on providing updated reporting guidelines across all surveillance levels. Stakeholders were also in consensus on the importance (90 %) of listing all the PC-NTDs in the reporting forms but there was non-consensus (55 %) on the feasibility of including all the diseases in the existing report forms. Further, participants agreed (88 %) on the need to ensure immediate reporting of PC-NTD cases to improve planned response actions. However, there was non-consensus on the achievability (66 %) of immediate case reporting on first detection. There was unanimity among stakeholders on the importance and feasibility of adopting electronic reporting tools to improve transmission of PC-NTDs surveillance data. Participants further reached consensus on the importance and feasibility of providing regular training on PC-NTDs reporting and allocating ample time for preparation and submission of surveillance reports.

Stakeholders had a converging opinion on the importance (98 %) and feasibility (93 %) of enhancing analysis of PC-NTD surveillance data. Participants also agreed on the need to conduct routine data analysis (84 %) and undertake periodic trend analysis (90 %) for PC-NTDs. However, there was lack of consensus on the feasibility of conducting regular data analysis (31 %) and periodic assessment of PC-NTDs trends (60 %).

*“We first need to improve the quality and quantity of data collected on PC-NTDs to be able to undertake routine analysis of these data”* (Participant 9).

Moreover, participants were in consensus on the importance and feasibility of enhancing training on data analysis, formulation of coherent action thresholds and provision of sufficient data analysis tools and equipment considering PC-NTDs.

There was consensus among stakeholders on the importance and feasibility of improving feedback specific to PC-NTDs and providing timely and regular feedback across all surveillance levels. In addition, participants agreed on the need and feasibility of adopting electronic feedback mechanisms and enhancing feedback on PC-NTDs to lower surveillance levels. Agreement was also reached on the importance and feasibility of updating outbreak preparedness and response protocols for all PC-NTDs and having well-constituted outbreak response teams at the sub-national level to adequately respond to PC-NTDs outbreaks. Participants further reached consensus on the need and feasibility of provision of sufficient emergency supplies in anticipation of probable outbreaks and providing frequent training on PC-NTDs epidemic preparedness and response.

### Surveillance support activities

Stakeholders reached consensus on the importance (100 %) and feasibility (91 %) of enhancing supervision of PC-NTDs surveillance activities at the sub-national levels. There was further agreement on the need to undertake regular supervision (92 %) of surveillance activities concerning PC-NTDs and heightening the frequency of supervisory visits (84 %) at the peripheral levels. However, participants disagreed (51 %) on the feasibility of consistently conducting supervision of surveillance activities and there was lack of consensus (22 %) on the possibility of increasing the frequency of supervisory visits at the lower levels.

*“Conducting regular supervision of surveillance activities requires resources for transportation and purchase of other materials which may not be feasible at this time due to other priority activities”* (Participant 12).

*“Due to the nature of the region which has very poor terrain, this pose a challenge to accessing some of the remote regions and conducting frequent supervision at the peripheral levels may not be practical”* (Participant 16).

Participants were in consensus on the necessity and feasibility to formulate supervisory schedules, train all health personnel on supervisory activities and establish supervisory teams capable of providing support supervision for PC-NTDs surveillance activities. Moreover, stakeholders agreed on the importance and feasibility of resource provision to support supervisory activities and involvement of the community levels to support supervision of PC-NTDs surveillance activities.

*“Support supervision should be strengthened especially at the peripheral levels with the involvement of the community units throughout the process”* (Participant 13).

*“Supervision of surveillance activities at the peripheral level requires the involvement of community health workers working within a functional community health unit”* (Participant 21).

Stakeholders had converging points of view on the importance and feasibility of enhanced and regular training on PC-NTDs surveillance activities. Participants further agreed on the need and feasibility of availing sufficient training materials and tools across all surveillance levels. However, participants ruled out consensus on the importance (70 %) of involving all health workers in training concerning surveillance activities for PC-NTDs.

*“Involving all the health workers in the region in surveillance training might not be cost effective and practical given the scarcity of resources set aside for conducting training”* (Participant 2).

*“This may not be feasible but we only need to ensure that an adequate number of staff are trained on surveillance of NTDs that are common in the region then they can pass on the knowledge gained to other health workers in their area of work”* (Participant 39).

There was unanimity on the importance (100 %) of limiting high turnover of trained surveillance staff. However, stakeholders lacked consensus (27 %) on the feasibility of retaining trained surveillance personnel across all surveillance levels.

*“Retention of health workers across surveillance levels may not be feasible since health workers are frequently transferred whenever there is need to adjust to given work circumstances”* (Participant 3).

*“This might not be feasible since staff progression and transfers normally happen regularly so it is better to train other staff to replace the ones transferred”* (Participant 37).

The stakeholders concurred on the need and feasibility of increased funding provision and logistical support for PC-NTDs surveillance activities and provision of electronic communication equipment for transmission of surveillance data. There was also consensus (82 %) on the importance of involving more health personnel in PC-NTDs surveillance activities but from a practical perspective, there was lack of consensus (40 %) among stakeholders on providing additional staff to be engaged in the surveillance activities. There was further lack of consensus on the importance (66 %) of increasing the number of designated surveillance focal persons.

*“This might lead to duplication of roles of the surveillance staff already in post, so the available staff should be able to handle all surveillance issues efficiently”* (Participant 38).

Additionally, stakeholders concurred on the need and feasibility of improving the surveillance data transmission and communication channels and providing reliable means of transport to facilitate surveillance activities.

### Surveillance attributes

Recommendation statements on improving simplicity, acceptability, stability and flexibility of the surveillance system concerning PC-NTDs were assessed in the two rounds. Findings showed that participants reached consensus regarding the importance and feasibility of simplifying existing reporting guidelines and forms, simplifying PC-NTDs case definitions for ease of application by health workers and simplifying methods of data collection and analysis of surveillance data. Further, there was unanimity among stakeholders on the need and feasibility for health managers across all levels to support PC-NTDs surveillance activities and for all health workers to consider PC-NTDs of public health importance in the endemic regions. In addition, participants agreed on the need and feasibility of promptly addressing challenges facing PC-NTDs surveillance activities and provision of adequate resources to support surveillance of the neglected tropical conditions. Stakeholders also reached consensus on the importance and feasibility of the existing surveillance system to adapt to reporting all PC-NTDs (100 %; 71 %) and for the system to adapt to changes in information needs regarding the diseases (100 %; 73 %).

Sensitivity analysis was undertaken with the assumption that participants with neutral responses were in agreement with the recommendation statements on both perspectives of importance and feasibility (Tables 4 and 5). Considering this scenario, findings indicated that stakeholders reached consensus on the importance (92 %) of availing case registers specific for PC-NTDs. Furthermore, there was consensus on the importance (92 %) of confirming PC-NTDs cases at the lower surveillance levels. Stakeholders further reached consensus on the importance (94 % & 88 %) of involving all health personnel in training on PC-NTDs surveillance activities and increasing the number of designated surveillance focal persons respectively, having assumed that those with neutral responses were in agreement with the statements. Further sensitivity analysis indicated that participants were in consensus on the feasibility of training all health workers on applying the available standard case definitions for PC-NTDs (91 %), increasing the number of laboratories at the lower levels (93 %), including all PC-NTDs in the existing reporting forms (93 %), ensuring immediate reporting (95 %) and undertaking periodic trend analysis concerning PC-NTDs (93 %). However, findings showed that stakeholders still lacked consensus on the feasibility of conducting routine data analysis for PC-NTDs surveillance data (51 %), increasing the frequency of supervisory visits at the lower surveillance levels (55 %) and retaining trained surveillance staff across all surveillance levels (47 %).

**Table 4:** Sensitivity analysis of non-consensus statements in the first round (N = 50)

Statements	Scenario 1: ‘Neither agree nor disagree’ excluded in analysis			Scenario 2: ‘Neither agree nor disagree’ included in analysis	
	**Consensus**	**Non-consensus**	**Neutral**	**Consensus**	**Non-consensus**
Availing case registers specific for registering PC-NTD cases is necessary	35 (70)*	4 (8)	11 (22)	46 (92)	4 (8)
All PC-NTDs need confirmation at the lower surveillance levels	33 (66)*	4 (8)	13 (26)	46 (92)	4 (8)
All health workers need to be involved in training on PC-NTDs surveillance activities	35 (70)*	3 (6)	12 (24)	47 (94)	3 (6)
Increasing the number of designated surveillance focal persons is required	33 (66)*	6 (12)	11 (22)	44 (88)	6 (12)

* - statements not achieving the > 70 % consensus threshold

**Table 5:** Sensitivity analysis of non-consensus statements in the second round (N = 45)

Statements	Scenario 1: ‘Neither agree nor disagree’ excluded in analysis			Scenario 2: ‘Neither agree nor disagree’ included in analysis	
	**Consensus**	**Non-consensus**	**Neutral**	**Consensus**	**Non-consensus**
It is feasible to train all health workers on the application of available PC-NTDs case definitions	31 (69)*	4 (9)	10 (22)	41 (91)	4 (9)
It is feasible to increase the number of laboratories at lower surveillance levels to improve PC-NTDs case confirmation capacity	16 (35)*	3 (7)	26 (58)	42 (93)	3 (7)
It is feasible to list all PC-NTDs in the existing reporting forms to improve surveillance data capture	25 (55)*	3 (7)	17 (38)	42 (93)	3 (7)
It is feasible to ensure immediate reporting of PC-NTD cases to improve planned response actions	30 (66)*	2 (4)	13 (29)	43 (95)	2 (4)
It is feasible for analysis of PC-NTDs surveillance data to be conducted on a routine-basis	14 (31)*	22 (49)	9 (20)	23 (51)*	22 (49)
It is feasible to undertake trend analysis of PC-NTDs reported cases periodically	27 (60)*	3 (7)	15 (33)	42 (93)	3 (7)
It is feasible to provide regular supervision of PC-NTDs surveillance activities undertaken at the lower levels	23 (51)*	2 (4)	20 (44)	43 (95)	2 (4)
It is feasible to increase the frequency of supervisory visits to the lower surveillance levels	10 (22)*	20 (45)	15 (33)	25 (55)*	20 (45)
It is feasible to retain trained surveillance staff across all surveillance levels	12 (27)*	24 (53)	9 (20)	21 (47)*	24 (53)
It is feasible to increase the number of health workers involved in PC-NTDs surveillance activities	18 (40)*	2 (4)	25 (56)	43 (96)	2 (4)

* - statements not achieving the > 70 % consensus threshold

## Discussion

Surveillance and response system improvement can only be guided by what is important, feasible, acceptable and tailored to the needs of system users. This Delphi survey identified critical and feasible recommendations for consideration and implementation by decision makers. The back and forth process of providing feedback based on an analysis of opinions from a group of participants enabled reaching consensus on the pertinent recommendations and the feasibility of their implementation [20]. The survey approach aimed to guide stakeholders’ decisions given the complexities and challenges of reaching an agreement on the most feasible strategies to improve surveillance activities concerning PC-NTDs. Findings identified convergence of opinion among participants on the importance and feasibility of all recommendations in six sub-domains relating to feedback, epidemic preparedness and response and on all the four surveillance attributes. Contrarily, participants failed to reach consensus on specific recommendations making up the remaining six sub-domains. Further sensitivity analysis indicated that participants considered all recommendation statements to be of importance. However, there was still non-consensus on the feasibility of implementing recommendations on routine data analysis, regular supervision and reducing trained staff turnover.

The study revealed that despite abundant evidence on the endemicity of NTDs amenable to chemoprophylaxis interventions in Kenya, there is limited data generated through the HIS to adequately guide evidence-based approaches for informed planning and implementation of sustainable interventions [33]. Findings indicated participant agreement on the need to update surveillance guidelines and standard case definitions. This call for deliberate efforts by the MOH to ensure standard case definitions (SCDs) for PC-NTDs are easy to understand and apply. However, surveillance guidelines bearing SCDs were updated recently in Kenya [34]. Notably, attempts to update surveillance guidelines are in line with outcomes from a previous forum, which recognised that containment of emergence and transmission of tropical conditions required use of updated SCDs for early and active case detection [13, 35]. Shifting emphasis from control to elimination entails utilisation of SCDs for prompt case detection and confirmation of suspected cases [2, 12]. On the other hand, limited laboratory capacity for case confirmation hinders meeting global targets for NTDs control and elimination since adequate laboratory data would guide decisions for effective implementation of interventions and monitoring progress towards halting disease transmission [36, 37]. In our study, participants agreement on strengthening PC-NTDs case confirmation capacities through adequately staffed and well equipped laboratories concurred with calls for improving case confirmation efforts regarding NTDs by increasing capacity of health facilities for routine diagnosis, increasing health workers laboratory skills and adequate provision of laboratory equipment [38].

Effective reporting systems limit the burden on public HIS in regions endemic of tropical diseases by informing the establishment of sentinel sites for assessing disease control interventions for targeted conditions [13]. This is in agreement with current findings indicating participant consensus on adoption of electronic tools for efficient reporting. Reliable reporting systems provide stakeholders with relevant information on the geographical distribution of NTD cases to conduct effective transmission assessment surveys [19]. Availability and adequacy of reporting tools ensures continuity and consistent reporting of surveillance data across surveillance levels [39, 40]. Furthermore, the Kenya MOH intends to formulate comprehensive NTDs reporting tools and advocates for joint reporting by both community drug distributors and health facility workers for further dissemination to the county, national and global levels [15]. However, in Sub Saharan regions, absence of clear indicators for specific NTDs in health management information systems (HMIS) hinders integration of the diseases into the existing reporting tools [38]. This could explain lack of consensus among participants on the feasibility for inclusion of all PC-NTDs in the reporting forms. Similarly, failure to reach consensus on the feasibility of PC-NTDs immediate reporting may be a consequence of emphasis on priority diseases including malaria, HIV/AIDS and tuberculosis and confining NTDs to conditions of low priority not requiring prompt action [41]. Specifically in Kenya, NTDs are categorised as “other diseases” in the summary report forms, which creates the perception that the conditions are of a lesser priority [14]. However, according to the Second Kenya National NTD Strategic Plan, deliberate efforts will be made to include NTDs in the HMIS register to capture all cases and ensure that they are immediately reportable upon detection [14].

Recommendations on enhanced data analysis for PC-NTDs are in line with efforts to ensuring public health surveillance and response systems institute rigorous data analysis and management tools that are vital in assessing attainment of elimination goals [13, 42, 43]. Similarly, studies in the African region have recommended the need for increased analysis of surveillance data through efforts to ensure routine data analysis and scaling up use of electronic reporting systems to improve accuracy of data entry and the resulting output from analysed data [44–46]. Increased data analysis facilitate assessment of disease trends over a given time period especially since reduced transmission leads to focalised case distribution that may inform implementation of targeted and appropriate responses [12, 47]. Our findings further identified the importance of enhancing training on data analysis, which is likely to provide health workers with the relevant skills and motivation to undertake routine data analysis. Findings from related studies indicate positive influence of training on improved reporting and analysis of surveillance data [27, 48, 49]. Moreover, provision of feedback within and across all health system levels is a vital undertaking within surveillance and response systems toward achieving disease elimination goals, which corresponds to participants’ consensus on enhanced feedback for PC-NTDs surveillance data [12]. However, focus for strengthening feedback mechanisms in developing countries has mainly been at the higher surveillance levels with less priority accorded to lower levels [50]. An effective feedback mechanism within existing surveillance systems limits information inundation and delayed transmission that may hinder initiation of prompt public health actions [13]. Furthermore, stakeholders’ consensus on increased feedback to lower levels would heighten community engagement in surveillance activities since community involvement and sensitisation is fundamental to achieving effective implementation of NTD interventions [38]. Similarly, an NTD control strategy in Kenya identifies the need for provision of joint feedback forums involving community health volunteers, community drug distributors and the wider community [15].

Having non-functional community health units that lack the capacity to coordinate supervisory activities in PC-NTDs endemic regions, may explain participants’ lack of agreement on the feasibility of undertaking regular supervision at peripheral levels. Essentially, engagement of community health personnel is critical to achieving effective implementation of NTD interventions [51]. The need to strengthen supervisory support for surveillance activities has been linked to improved disease detection and notification, utilisation of updated surveillance information and overall improved performance of surveillance systems [6, 50, 52]. Furthermore, there are calls to ensure that supportive supervision is provided for all diseases being reported in Kenya [15]. On the other hand, training of health workers regarding surveillance activities is linked to improved reporting performance and overall strengthening of national disease surveillance systems [48]. Similar to our findings, there was agreement among participants on the importance of surveillance training focused on PC-NTDs, enhancing training frequency of health personnel and adequate provision of training materials and tools. However, participants held the view that involvement of health workers of all cadres in surveillance training might not be feasible due to the cost implications of facilitating numerous training activities. However, most regional levels in African countries provide human, technical and financial support for training of trainers, which facilitates cascade training and adoption of on-the-job training and pre-service training mechanisms [48]. Nevertheless, participants were not in consensus with feasibility of expanding the health workforce responsible for disease surveillance and response activities at the sub-national level. This could be attributed to scarcity of resources to increase the number of surveillance staff and the general shortage of health personnel [14]. Therefore, the sub-national levels ought to leverage on efficient use of the existing workforce and other surveillance resources. A unified approach to utilise the available resources is a key principle of the IDSR framework [4]. Furthermore, integrated efforts to control NTDs are encouraged to augment utilisation of the limited resources [14]. The geographical overlap and co-endemicity of PC-NTDs rationalises the need for integration of surveillance and control efforts [15, 41].

## Strengths and limitations

The study approach enabled an extensive assessment on the cardinal and feasible recommendations based on consensus among concerned stakeholders, which are pertinent to improving disease surveillance and response to PC-NTDs in Kenya. First, the survey focused on assessing stakeholders’ perceptions at the sub-national level, because this level provides first contact to healthcare services for communities living in NTD endemic regions. However, subsequent surveys could further assess stakeholders’ perspectives at the national surveillance level, since decisions made at the national level may influence implementation of feasible recommendations sub-nationally. Secondly, important recommendations that lacked consensus on feasibility for implementation in the second round were not subjected to further inquiry. However, further subsequent investigations considering these recommendations will aid in ensuring optimal performance of the overall surveillance system. Thirdly, due to the existing COVID-19 pandemic while conducting the study, it required strict adherence to physical distancing measures between the interviewer and study participants. Therefore, the Delphi consensus survey that can be conducted electronically was an appropriate methodological approach for collating stakeholders’ views and assessing consensus among the participants. However, criticisms regarding the validity of the Delphi technique relate to a form of bias in its conduct, in that the consensus reached can be influenced by the way the researcher administers the data collection tool to the group of experts [25]. Additionally, as a common practice in most Delphi studies, the use of interviews to obtain expert opinions in the first rounds may be biased by stronger opinions coming from highly regarded experts [25]. However, in the current study, both Delphi study rounds utilised electronic self-administered likert-type tools. Use of electronic data collection instruments provided an equal chance for participation avoiding bias resulting from participants with dominant opinions. Furthermore, the identity of the participants enrolled in the two analytic rounds were kept anonymous to mitigate biasness during analysis. The size and composition of the group of stakeholders may not represent all decision makers at the sub-national level and may not be representative of all PC-NTD endemic regions in Kenya, hence reducing generalisability of the findings. However, the minimum threshold of at least twelve experts in a Delphi survey was met and having more participants with limited interaction increases reliability of group judgement [53]. Further, the sensitivity analysis undertaken in the current study was biased towards the assumption that participants with neutral responses were in agreement with the recommendation statements. Lastly, there were a limited number of similar studies to make sufficient comparisons with our study findings. However, this study provides insights into feasible actions to improve PC-NTDs surveillance activities and should inform further research in other NTD endemic countries.

## Conclusions

Sustained efforts to control and eliminate PC-NTDs are hinged on optimal functioning of HIS. Essentially, surveillance and response activities form part of the definitive phase to achieving NTDs elimination through halted disease transmission [13]. Achieving consensus among stakeholders on the implementable recommendations will aid in identification of priority actions at the sub-national levels. However, non-consensus recommendations relating to PC-NTDs case confirmation capacities, routine data analysis, increased supervision, trained surveillance staff turnover and adequacy of surveillance personnel may require further consultations with concerned stakeholders to ensure implementation of effective policies to achieve the intended outcomes. NTDs agenda setting requires consolidation of all stakeholders’ views and further formulation of NTDs policies relies on adequate partnership and coordination at the sub-national and national levels of the health system [22]. Lack of reliable HIS will limit data utilisation resulting to sub-optimal planning and coordination of NTD programme activities. Therefore, stakeholders’ consensus and adoption of feasible recommendations identifies key opportunities for policy refinement and decision-making to strengthen NTDs surveillance and response activities at the sub-national level.

No funding received.

## Supplementary information

**Additional file 1**

**Additional file 2**

## Data Availability

The datasets generated and/or analysed to assess the surveillance system attributes are not publicly available due to the need to keep the identities of participants confidential as they granted consent to be enrolled in the study on the basis of remaining anonymous, but are available from the corresponding author on reasonable request.

## Abbreviations

BTS: Breaking Transmission Strategy
CDC: Centres for Disease Control and Prevention
CHMC: County Health Management Committee
COVID-19: Coronavirus disease
DEC: Diethylcarbamizine
ESPEN: Expanded Special Project for Elimination of Neglected Tropical Diseases
HIS: Health Information Systems
HMIS: Health Management Information Systems
IDSR: Integrated Diseases Surveillance and Response
IQR: Inter Quartile Range
IREC: Institutional Research and Ethics Committee
LF: Lymphatic Filariasis
MOH: Ministry of Health
NACOSTI: National Commission for Science, Technology and Innovation
NTDs: Neglected Tropical Diseases
PC-NTDs: Preventive Chemotherapy Neglected Tropical Diseases
PROSPERO: International Prospective Register of Systematic Reviews
SCHMC: Sub-County Health Management Committee
STH: Soil Transmitted Helminthiasis
USA: United States of America
WHO: World Health Organization
