# Supplementary material for: Feasibility of implementing recommendations to improve neglected tropical diseases surveillance and response in Kenya: a modified Delphi study
Source: BMC Health Serv Res. 2021 Oct 1;21:1034. doi: 10.1186/s12913-021-07075-y (PMC8485576; doi:10.1186/s12913-021-07075-y)
Supplement: Supplementary file 1 — Additional file 1 [file 12913_2021_7075_MOESM1_ESM.docx]

## Supplementary file 1 – Delphi study questionnaire (Round 1)

| **PARTICIPANT INFORMATION** | | | | | | | | |
| --- | --- | --- | --- | --- | --- | --- | --- | --- |
| Region | | □ Baringo County  □ West Pokot County  □ Narok County  □ Kwale County  □ Kilifi County  □ Embu County  □ Kitui County | | | | | | |
| Demographic characteristics | | Age | | | □ 18-30  □ 31-40  □ 41-50  □ >50 | | | |
|  |  | Sex | | | □ Male  □ Female | | | |
| Current work designation | | ……………………………………………………………………. | | | | | | |
| Years of experience in your current work designation | | ………………………… years | | | | | | |
| **(Tick (√) in the most appropriate box & please complete the “comments” section provided in each sub-category)** | | | | | | | | |
| **CORE FUNCTIONS**  **1) Case detection, registration and confirmation** | | | | | | | | |
|  | ***Strongly Disagree*** | | ***Disagree*** | ***Neither Agree or Disagree*** | | ***Agree*** | ***Strongly***  ***Agree*** | ***Don’t***  ***Know*** |
| **Q1.** Need to update surveillance guidelines currently in use at the sub-national level |  | |  |  | |  |  |  |
| **Q2.** Need to update the available PC-NTDs case definitions currently in use at the sub-national level |  | |  |  | |  |  |  |
| **Q3.** Training is required on practical application of the available PC-NTDs case definitions |  | |  |  | |  |  |  |
| **Q4.** Availing case registers specific for registering PC-NTD cases is necessary |  | |  |  | |  |  |  |
| **Q5.** All PC-NTDs need confirmation at the lower surveillance levels |  | |  |  | |  |  |  |
| **Q6.** Increased number of laboratories at lower surveillance levels is needed to improve PC-NTDs case confirmation capacity |  | |  |  | |  |  |  |
| **Q7.**Need to properly equip laboratories at the health facility level to improve PC-NTDs case confirmation |  | |  |  | |  |  |  |
| **Q8.** Need to provide an adequate number of skilled laboratory health personnel for effective confirmation of PC-NTDs |  | |  |  | |  |  |  |
| **Comments:** | | | | | | | | |
| **2) Reporting** | | | | | | | | |
|  | ***Strongly Disagree*** | | ***Disagree*** | ***Neither Agree or Disagree*** | | ***Agree*** | ***Strongly***  ***Agree*** | ***Don’t***  ***Know*** |
| **Q9.** Need to ensure reporting forms are always readily available in all surveillance levels |  | |  |  | |  |  |  |
| **Q10.** Need to avail updated reporting guidelines at the sub-national level |  | |  |  | |  |  |  |
| **Q11.** Need to list all PC-NTDs in the existing reporting forms to improve surveillance data capture |  | |  |  | |  |  |  |
| **Q12.** Immediate reporting of PC-NTD cases is required to improve planned response actions |  | |  |  | |  |  |  |
| **Q13.** Need to adopt electronic reporting tools to improve transmission of PC-NTDs surveillance data to the next level |  | |  |  | |  |  |  |
| **Q14.** Frequent training on reporting PC-NTDs using existing reporting forms is required |  | |  |  | |  |  |  |
| **Q15.** Need to allocate adequate time for surveillance reports preparation and submission to the next levels |  | |  |  | |  |  |  |
| **Comments:** | | | | | | | | |
| **3. Data analysis** | | | | | | | | |
|  | ***Strongly Disagree*** | | ***Disagree*** | ***Neither Agree or Disagree*** | | ***Agree*** | ***Strongly***  ***Agree*** | ***Don’t***  ***Know*** |
| **Q16.** Need to enhance PC-NTDs surveillance data analysis at the sub-national level |  | |  |  | |  |  |  |
| **Q17.** Analysis of PC-NTDs surveillance data should be conducted on a routine-basis |  | |  |  | |  |  |  |
| **Q18.** Trend analysis of PC-NTDs reported cases should be undertaken periodically |  | |  |  | |  |  |  |
| **Q19.** Enhanced training on PC-NTDs surveillance data analysis is required |  | |  |  | |  |  |  |
| **Q20.** Clearly formulated PC-NTDs action thresholds are required |  | |  |  | |  |  |  |
| **Q21.** Need to provide adequate data analysis tools and equipment |  | |  |  | |  |  |  |
| **Comments:** | | | | | | | | |
| **4. Feedback** | | | | | | | | |
|  | ***Strongly Disagree*** | | ***Disagree*** | ***Neither Agree or Disagree*** | | ***Agree*** | ***Strongly***  ***Agree*** | ***Don’t***  ***Know*** |
| **Q22.** Need to improve feedback on PC-NTDs surveillance data at the sub-national level |  | |  |  | |  |  |  |
| **Q23.** Need for timely feedback on PC-NTDs surveillance data reported to the next level |  | |  |  | |  |  |  |
| **Q24.** Regular feedback on reported PC-NTDs surveillance data is required |  | |  |  | |  |  |  |
| **Q25.** Need to adapt improved electronic feedback mechanisms |  | |  |  | |  |  |  |
| **Q26.** Increased feedback on PC-NTDs to lower surveillance levels is required |  | |  |  | |  |  |  |
| **Comments:** | | | | | | | | |
| **5. Epidemic preparedness and response** | | | | | | | | |
|  | ***Strongly Disagree*** | | ***Disagree*** | ***Neither Agree or Disagree*** | | ***Agree*** | ***Strongly***  ***Agree*** | ***Don’t***  ***Know*** |
| **Q27.** Updated PC-NTDs outbreak preparedness and response protocols are required |  | |  |  | |  |  |  |
| **Q28.** Need for outbreak response teams to be well-constituted to respond to probable PC-NTDs outbreaks |  | |  |  | |  |  |  |
| **Q29.** Need for emergency supplies to adequately respond to probable PC-NTDs outbreaks |  | |  |  | |  |  |  |
| **Q30.** Regular training on PC-NTDs outbreak preparedness and response is required |  | |  |  | |  |  |  |
| **Comments:** | | | | | | | | |
| **SUPPORT FUNCTIONS**  **1) Supervision** | | | | | | | | |
|  | ***Strongly Disagree*** | | ***Disagree*** | ***Neither Agree or Disagree*** | | ***Agree*** | ***Strongly***  ***Agree*** | ***Don’t***  ***Know*** |
| **Q31.** Need to enhance supervision of PC-NTDs surveillance activities at the sub-national level |  | |  |  | |  |  |  |
| **Q32.** Regular supervision of PC-NTDs surveillance activities undertaken at the lower levels is required |  | |  |  | |  |  |  |
| **Q33.** Formulation of supervisory schedules for PC-NTDs surveillance activities is necessary |  | |  |  | |  |  |  |
| **Q34.** Increased frequency of supervisory visits at the lower surveillance levels is required |  | |  |  | |  |  |  |
| **Q35.** Training and sensitisation of all health workers regarding supervisory activities is required |  | |  |  | |  |  |  |
| **Q36.** Need for properly constituted supervisory teams to adequately supervise PC-NTDs surveillance activities |  | |  |  | |  |  |  |
| **Q37.** Need for adequate resource provision to support supervision of PC-NTDs surveillance activities |  | |  |  | |  |  |  |
| **Q38.** Need for increased participation of the community levels to support supervision of PC-NTDs surveillance activities |  | |  |  | |  |  |  |
| **Comments:** | | | | | | | | |
| **2) Training** | | | | | | | | |
|  | ***Strongly Disagree*** | | ***Disagree*** | ***Neither Agree or Disagree*** | | ***Agree*** | ***Strongly***  ***Agree*** | ***Don’t***  ***Know*** |
| **Q39.** Need to improve training on disease surveillance at sub-national level to focus on PC-NTDs |  | |  |  | |  |  |  |
| **Q40.** Regular training specifically on PC-NTDs surveillance activities is necessary |  | |  |  | |  |  |  |
| **Q41.** All health workers need to be involved in training on PC-NTDs surveillance activities |  | |  |  | |  |  |  |
| **Q42.** Availing adequate training materials and equipment is necessary across all surveillance levels |  | |  |  | |  |  |  |
| **Q43.** Need to retain trained surveillance staff across all surveillance levels |  | |  |  | |  |  |  |
| **Comments:** | | | | | | | | |
| **3) Resources** | | | | | | | | |
|  | ***Strongly Disagree*** | | ***Disagree*** | ***Neither Agree or Disagree*** | | ***Agree*** | ***Strongly***  ***Agree*** | ***Don’t***  ***Know*** |
| **Q44.** Increased funding is required to support PC-NTDs surveillance activities |  | |  |  | |  |  |  |
| **Q45.** Availing electronic communication equipment for transmission of PC-NTDs surveillance data is required |  | |  |  | |  |  |  |
| **Q46.** Improved transport and logistical support is necessary to facilitate PC-NTDs surveillance activities |  | |  |  | |  |  |  |
| **Q47.** Increasing the number of health workers involved in PC-NTDs surveillance activities is necessary |  | |  |  | |  |  |  |
| **Q48.** Increasing the number of designated surveillance focal persons is required |  | |  |  | |  |  |  |
| **Q49.** Need for improved telecommunication channels to support transmission of surveillance data |  | |  |  | |  |  |  |
| **Q50.** Need for improved means of transportation to facilitate surveillance activities |  | |  |  | |  |  |  |
| **Comments:** | | | | | | | | |
| **SURVEILLANCE ATTRIBUTES**  **1) Simplicity** | | | | | | | | |
|  | ***Strongly Disagree*** | | ***Disagree*** | ***Neither Agree or Disagree*** | | ***Agree*** | ***Strongly***  ***Agree*** | ***Don’t***  ***Know*** |
| **Q51.** Simplification of existing guidelines for completing reporting forms is required |  | |  |  | |  |  |  |
| **Q52.** Need to simplify available forms to ease reporting of PC-NTDs |  | |  |  | |  |  |  |
| **Q53.** Need to simplify PC-NTDs case definitions to ease application |  | |  |  | |  |  |  |
| **Q54.** Need to simplify methods for PC-NTDs surveillance data collection and analysis |  | |  |  | |  |  |  |
| **Comments:** | | | | | | | | |
| **2) Acceptability** | | | | | | | | |
|  | ***Strongly Disagree*** | | ***Disagree*** | ***Neither Agree or Disagree*** | | ***Agree*** | ***Strongly***  ***Agree*** | ***Don’t***  ***Know*** |
| **Q55.** Need for the health managers to support PC-NTDs surveillance activities in the region |  | |  |  | |  |  |  |
| **Q56.** PC-NTDs need to be considered of public health importance in the region |  | |  |  | |  |  |  |
| **Comments:** | | | | | | | | |
| **3) Stability** | | | | | | | | |
|  | ***Strongly Disagree*** | | ***Disagree*** | ***Neither Agree or Disagree*** | | ***Agree*** | ***Strongly***  ***Agree*** | ***Don’t***  ***Know*** |
| **Q57.** Need for challenges facing PC-NTDs surveillance activities to be addressed with minimal delays |  | |  |  | |  |  |  |
| **Q58.** Sufficient resources to support PC-NTDs surveillance activities are required |  | |  |  | |  |  |  |
| **Comments:** | | | | | | | | |
| **4) Flexibility** | | | | | | | | |
|  | ***Strongly Disagree*** | | ***Disagree*** | ***Neither Agree or Disagree*** | | ***Agree*** | ***Strongly***  ***Agree*** | ***Don’t***  ***Know*** |
| **Q59.** Need for existing surveillance systems to be well adapted to reporting all PC-NTDs in the region |  | |  |  | |  |  |  |
| **Q60.** Existing surveillance systems need to adapt easily to changes in PC-NTDs information needs |  | |  |  | |  |  |  |
| **Comments:** | | | | | | | | |
