# Supplementary material for: Feasibility of implementing recommendations to improve neglected tropical diseases surveillance and response in Kenya: a modified Delphi study
Source: BMC Health Serv Res. 2021 Oct 1;21:1034. doi: 10.1186/s12913-021-07075-y (PMC8485576; doi:10.1186/s12913-021-07075-y)
Supplement: Supplementary file 2 — Additional file 2 [file 12913_2021_7075_MOESM2_ESM.docx]

## Supplementary file 2 – Delphi study questionnaire (Round 2)

| **PARTICIPANT INFORMATION** | | | | | | | |
| --- | --- | --- | --- | --- | --- | --- | --- |
| Region | | □ Baringo County  □ West Pokot County  □ Narok County  □ Kwale County  □ Kilifi County  □ Embu County  □ Kitui County | | | | | |
| Demographic characteristics | | Age | | □ 18-30  □ 31-40  □ 41-50  □ >50 | | | |
|  |  | Sex | | □ Male  □ Female | | | |
| Current work designation | | ……………………………………………………………………. | | | | | |
| Years of experience in your current work designation | | ………………………… years | | | | | |
| **(Tick (√) in the most appropriate box & please complete the “comments” section provided in each sub-category)** | | | | | | | |
| **CORE FUNCTIONS**  **1) Case detection, registration and confirmation** | | | | | | | |
|  | ***Strongly Disagree*** | | ***Disagree*** | ***Neither Agree or Disagree*** | ***Agree*** | ***Strongly***  ***Agree*** | ***Don’t***  ***Know*** |
| **Q1.** It is feasible to update surveillance guidelines currently in use at the sub-national level |  | |  |  |  |  |  |
| **Q2.** It is feasible to update the available PC-NTDs case definitions currently in use at the sub-national level |  | |  |  |  |  |  |
| **Q3.** It is feasible to train all health workers on the application of available PC-NTDs case definitions |  | |  |  |  |  |  |
| **Q4.** It is feasible to increase the number of laboratories at lower surveillance levels to improve PC-NTDs case confirmation capacity |  | |  |  |  |  |  |
| **Q5.** It is feasible to fully equip laboratories at the health facility level to improve PC-NTDs case confirmation |  | |  |  |  |  |  |
| **Q6.** It is feasible to provide an adequate number of skilled laboratory health personnel for effective confirmation of PC-NTDs |  | |  |  |  |  |  |
| **Comments:** | | | | | | | |
| **2) Reporting** | | | | | | | |
|  | ***Strongly Disagree*** | | ***Disagree*** | ***Neither Agree or Disagree*** | ***Agree*** | ***Strongly***  ***Agree*** | ***Don’t***  ***Know*** |
| **Q7.** It is feasible to ensure reporting forms are always readily available in all surveillance levels |  | |  |  |  |  |  |
| **Q8.** It is feasible to avail updated reporting guidelines at the sub-national level |  | |  |  |  |  |  |
| **Q9.** It is feasible to list all PC-NTDs in the existing reporting forms to improve surveillance data capture |  | |  |  |  |  |  |
| **Q10.** It is feasible to ensure immediate reporting of PC-NTD cases to improve planned response actions |  | |  |  |  |  |  |
| **Q11.** It is feasible to adopt electronic reporting tools to improve transmission of PC-NTDs surveillance data to the next level |  | |  |  |  |  |  |
| **Q12.** It is feasible to offer frequent training on reporting PC-NTDs using existing reporting forms |  | |  |  |  |  |  |
| **Q13.** It is feasible to allocate adequate time for surveillance reports preparation and submission to the next levels |  | |  |  |  |  |  |
| **Comments:** | | | | | | | |
| **3. Data analysis** | | | | | | | |
|  | ***Strongly Disagree*** | | ***Disagree*** | ***Neither Agree or Disagree*** | ***Agree*** | ***Strongly***  ***Agree*** | ***Don’t***  ***Know*** |
| **Q14.** It is feasible to enhance PC-NTDs surveillance data analysis at the sub-national level |  | |  |  |  |  |  |
| **Q15.** It is feasible for analysis of PC-NTDs surveillance data to be conducted on a routine-basis |  | |  |  |  |  |  |
| **Q16.** It is feasible to undertake trend analysis of PC-  NTDs reported cases periodically |  | |  |  |  |  |  |
| **Q17.** It feasible to enhance training on PC-NTDs surveillance data analysis |  | |  |  |  |  |  |
| **Q18.** It is feasible to have clearly formulated PC-NTDs action thresholds |  | |  |  |  |  |  |
| **Q19.** It is feasible to provide adequate data analysis tools and equipment to all surveillance levels |  | |  |  |  |  |  |
| **Comments:** | | | | | | | |
| **4. Feedback** | | | | | | | |
|  | ***Strongly Disagree*** | | ***Disagree*** | ***Neither Agree or Disagree*** | ***Agree*** | ***Strongly***  ***Agree*** | ***Don’t***  ***Know*** |
| **Q20.** It is feasible to improve feedback on PC-NTDs surveillance data at the sub-national level |  | |  |  |  |  |  |
| **Q21.** It is feasible to provide timely feedback on PC-NTDs surveillance data reported to the next level |  | |  |  |  |  |  |
| **Q22.** It is feasible to provide regular feedback on reported PC-NTDs surveillance data |  | |  |  |  |  |  |
| **Q23.** It is feasible to adapt improved electronic feedback mechanisms |  | |  |  |  |  |  |
| **Q24.** It is feasible to increase feedback on PC-NTDs to lower surveillance levels |  | |  |  |  |  |  |
| **Comments:** | | | | | | | |
| **5. Epidemic preparedness and response** | | | | | | | |
|  | ***Strongly Disagree*** | | ***Disagree*** | ***Neither Agree or Disagree*** | ***Agree*** | ***Strongly***  ***Agree*** | ***Don’t***  ***Know*** |
| **Q25.** It is feasible to provide all surveillance levels with updated PC-NTDs outbreak preparedness and response protocols |  | |  |  |  |  |  |
| **Q26.** It is feasible to have well constituted outbreak response teams to respond to probable PC-NTDs outbreaks |  | |  |  |  |  |  |
| **Q27.** It is feasible to provide adequate emergency supplies to respond to probable PC-NTDs outbreaks |  | |  |  |  |  |  |
| **Q28.** It is feasible to provide regular training on PC-NTDs outbreak preparedness and response |  | |  |  |  |  |  |
| **Comments:** | | | | | | | |
| **SUPPORT FUNCTIONS**  **1) Supervision** | | | | | | | |
|  | ***Strongly Disagree*** | | ***Disagree*** | ***Neither Agree or Disagree*** | ***Agree*** | ***Strongly***  ***Agree*** | ***Don’t***  ***Know*** |
| **Q29.** It is feasible to enhance supervision of PC-NTDs surveillance activities at the sub-national level |  | |  |  |  |  |  |
| **Q30.** It is feasible to provide regular supervision of PC-NTDs surveillance activities undertaken at the lower levels |  | |  |  |  |  |  |
| **Q31.** It is feasible to formulate supervisory schedules for PC-NTDs surveillance activities |  | |  |  |  |  |  |
| **Q32.** It is feasible to increase the frequency of supervisory visits to the lower surveillance levels |  | |  |  |  |  |  |
| **Q33.** It is feasible to train and sensitise all health workers regarding supervisory activities |  | |  |  |  |  |  |
| **Q34.** It is feasible to properly constitute supervisory teams to adequately supervise PC-NTDs surveillance activities |  | |  |  |  |  |  |
| **Q35.** It is feasible to ensure there is adequate resource provision to support supervision of PC-NTDs surveillance activities |  | |  |  |  |  |  |
| **Q36.** It is feasible to increase community participation to support supervision of PC-NTDs surveillance activities |  | |  |  |  |  |  |
| **Comments:** | | | | | | | |
| **2) Training** | | | | | | | |
|  | ***Strongly Disagree*** | | ***Disagree*** | ***Neither Agree or Disagree*** | ***Agree*** | ***Strongly***  ***Agree*** | ***Don’t***  ***Know*** |
| **Q37.** It is feasible to improve PC-NTDs surveillance trainings conducted at the sub-national level |  | |  |  |  |  |  |
| **Q38.** It is feasible to provide regular training specifically on PC-NTDs surveillance activities |  | |  |  |  |  |  |
| **Q39.** It is feasible to avail adequate training materials and equipment across all surveillance levels |  | |  |  |  |  |  |
| **Q40.** It is feasible to retain trained surveillance staff across all surveillance levels |  | |  |  |  |  |  |
| **Comments:** | | | | | | | |
| **3) Resources** | | | | | | | |
|  | ***Strongly Disagree*** | | ***Disagree*** | ***Neither Agree or Disagree*** | ***Agree*** | ***Strongly***  ***Agree*** | ***Don’t***  ***Know*** |
| **Q41.** It is feasible to increase funding to support PC-NTDs surveillance activities |  | |  |  |  |  |  |
| **Q42.** It is feasible to provide electronic communication equipment for transmission of PC-NTDs surveillance data |  | |  |  |  |  |  |
| **Q43.** It is feasible to improve transport and logistical support to facilitate PC-NTDs surveillance activities |  | |  |  |  |  |  |
| **Q44.** It is feasible to increase the number of health workers involved in PC-NTDs surveillance activities |  | |  |  |  |  |  |
| **Q45.** It is feasible to improve telecommunication channels to support transmission of surveillance data |  | |  |  |  |  |  |
| **Q46.** It is feasible to improve means of transportation to facilitate surveillance activities |  | |  |  |  |  |  |
| **Comments:** | | | | | | | |
| **SURVEILLANCE ATTRIBUTES**  **1) Simplicity** | | | | | | | |
|  | ***Strongly Disagree*** | | ***Disagree*** | ***Neither Agree or Disagree*** | ***Agree*** | ***Strongly***  ***Agree*** | ***Don’t***  ***Know*** |
| **Q47.** It is feasible to simplify existing guidelines for completing reporting forms |  | |  |  |  |  |  |
| **Q48.** It is feasible to simplify available forms to ease reporting of PC-NTDs |  | |  |  |  |  |  |
| **Q49.** It is feasible to simplify PC-NTDs case definitions to ease application |  | |  |  |  |  |  |
| **Q50.** It is feasible to simplify methods for PC-NTDs surveillance data collection and analysis |  | |  |  |  |  |  |
| **Comments:** | | | | | | | |
| **2) Acceptability** | | | | | | | |
|  | ***Strongly Disagree*** | | ***Disagree*** | ***Neither Agree or Disagree*** | ***Agree*** | ***Strongly***  ***Agree*** | ***Don’t***  ***Know*** |
| **Q51.** It is feasible for the health managers to support PC-NTDs surveillance activities in the region |  | |  |  |  |  |  |
| **Q52.** It is feasible to influence health workers’ perceptions on the public health importance of PC-NTDs in the region |  | |  |  |  |  |  |
| **Comments:** | | | | | | | |
| **3) Stability** | | | | | | | |
|  | ***Strongly Disagree*** | | ***Disagree*** | ***Neither Agree or Disagree*** | ***Agree*** | ***Strongly***  ***Agree*** | ***Don’t***  ***Know*** |
| **Q53.** It is feasible to address challenges facing PC-NTDs surveillance activities with minimal delays |  | |  |  |  |  |  |
| **Q54.** It is feasible to avail sufficient resources to support PC-NTDs surveillance activities |  | |  |  |  |  |  |
| **Comments:** | | | | | | | |
| **4) Flexibility** | | | | | | | |
|  | ***Strongly Disagree*** | | ***Disagree*** | ***Neither Agree or Disagree*** | ***Agree*** | ***Strongly***  ***Agree*** | ***Don’t***  ***Know*** |
| **Q55.** It is feasible for the existing surveillance systems to be well adapted to reporting all PC-NTDs in the region |  | |  |  |  |  |  |
| **Q56.** It is feasible for the existing surveillance systems to adapt easily to changes in PC-NTDs information needs |  | |  |  |  |  |  |
| **Comments:** | | | | | | | |
